# Mortality Time-Trends of Different Cardiovascular Diseases in a Practically Extinct Cohort of Italian Middle-Aged Men Followed-Up for 61 Years: A Possible Etiological Explanation?

**DOI:** 10.3390/jcdd11030094

**Published:** 2024-03-21

**Authors:** Paolo Emilio Puddu, Paolo Piras, Alessandro Menotti

**Affiliations:** 1 Association for Cardiac Research, 00182 Rome, Italy; amenotti2@gmail.com; 2 EA 4650, Signalisation, Électrophysiologie et Imagerie des Lésions d’Ischémie Reperfusion Myocardique, Université de Normandie, 14000 Caen, France; 3 Department of Structural Engineering, Sapienza University of Rome, 00185 Rome, Italy; paolo.piras@uniroma3.it

**Keywords:** lifetime CVD mortality, risk factors, CVD natural history

## Abstract

Purpose. To study a male Italian cohort (initially aged 40–59, n = 1712) during 61 years and the natural history of major CVD mortality categories including coronary heart disease (CHD), stroke and other heart diseases of uncertain etiology (HDUE), including congestive heart failure) along with their risk factor relationships. Methods and Results. Cox models were run with 12 covariates as possible predictors measured at entry to the study. About 93% of all CVD deaths were covered by the three major groups selected here (N = 751): 37.4% of them were diagnosed as CHD, 30.6% as stroke and 28.5% as HDUE. CHD declined in the last 20 years of follow-up, while a sharp increase in HDUE mortality was seen. Baseline mean levels of serum cholesterol were 209.6, 204.2 and 198.0 mg/dL, respectively, for CHD, stroke and HDUE deaths: the multivariable coefficients of serum cholesterol were positive and significant for CHD (*p* < 0.0001), and stroke (*p* = 0.0203) and not significant for HDUE (*p* = 0.3467). In Fine–Gray models, the algebraic signs of cholesterol coefficients were opposite for CHD versus the other mortality categories (t = 3.13). The predictive performances of remaining risk factors were varied whereas that of Cox models was not very good, probably due to the attrition phenomenon and possible competing risks. Conclusion. Large differences in natural history and risk factors were found comparing the three CVD conditions, potentially indicating different etiologies and pointing to the need of not mixing them up in a grouped CVD category.

## 1. Introduction

The cohorts of middle-aged men belonging to the Italian Rural Areas (IRA) of the Seven Countries Study of Cardiovascular Diseases (SCS) have reached a practical extinction after 61 years of follow-up since only three men (out of the initial 1712 participants) are still alive. This prompted the idea to describe the time-trends of mortality and other characteristics across major groups of cardiovascular diseases (CVDs) that occurred during the lifespan of those men. We also aimed to explain potential differences by considering the influence of risk factors associated with those CVD groups.

In previous analyses on shorter follow-up periods [1,2,3], but also on different cohorts of the SCS [4,5,6], we focused on coronary heart disease (CHD) versus a group of ill-defined heart diseases that we called heart diseases of uncertain etiology (HDUE, including congestive heart failure) and large differences from CHD were observed. For the purpose of the present analysis, the groups of CHD and HDUE were contrasted to stroke being another major component of CVDs. It is important to note that the current existing literature does not concentrate on these group differences, apart those of a clinically defined group of congestive heart failure (CHF), so that discussing outcomes in a practically extinct population-based series becomes quite critical for the time-trend understanding and the natural history of these conditions comparatively.

## 2. Material and Methods

### 2.1. Study Population

The analysis was run in the pool of two cohorts of middle-aged men (40–59 years old) belonging to the IRA of the Seven Countries Study of Cardiovascular Diseases (SCS). The defined samples included a total of 1735 men and 1712 were examined at the start in 1960 (participation rate of 98.7%). More information is available elsewhere [7].

### 2.2. Mortality Data

Follow-up for life status, mortality and causes of death was extended for 61 years and only one man was lost to follow-up after 50 years when he was aged 91 years, while three men were still alive. Causes of death were allocated by reviewing and combining information from death certificates, interim examinations, hospital and medical records, interviews with physicians and relatives of the deceased and any other witnesses of fatal events. Causes of death were determined by a single coder (AM) following defined criteria, and employing the 8th Revision of the WHO-ICD (ICD-8) [8]. In the presence of multiple causes and uncertainty about the principal cause, a hierarchical preference was adopted with violence, cancers, coronary heart disease, stroke and other, in that order [9].

The end-points of this analysis were the following groups of cardiovascular diseases:

(1) CHD including cases of myocardial infarction, acute ischemic attacks and sudden coronary death occurring within 2 h from onset of symptoms, after the exclusion of other possible causes (ICD-8 codes 410, 411, 412, 413, 795); cases with only a mention or evidence of chronic coronary heart diseases (part of ICD-8 code 412) or classified as other ischemic heart disease (ICD-8 code 414) were not included in this group for reasons given elsewhere [1,2], but healed myocardial infarction was retained in this group;
(2) Stroke including any type of cerebrovascular diseases (ICD-8 codes 430–438);
(3) Among other types of CVD, we selected and created a large subgroup of heart disease of uncertain etiology (HDUE), including symptomatic heart disease (ICD-8 code 427) (thus CHF of 62% of this group), hypertensive heart disease, usually poorly documented (ICD-8 codes 400–404) (13%) and cases vaguely quoted as chronic or other types of coronary heart disease, (ICD code 412, 414) in the absence of any evidence of typical coronary syndromes (25%); usually they were manifested as heart failure, arrhythmia and blocks.

All other rare or etiologically defined cardiovascular disease were not included in this analysis.

### 2.3. Risk Factors

A large number of potential risk factors were measured but for the purpose of this analysis only 12 were selected, to be conservative and taking into account their role shown in previous analyses run on shorter follow-up periods [7].

These were: (a) age in years, approximated to the nearest birthday; (b) family history of heart attacks, among close relatives (0 = no; 1 = yes); (c) cigarettes smoked on average per day (n/day), which was adopted as a metric since preliminary analyses showed that in the long run ex-smoker had a risk rather similar to the non-smokers (both groups being classified with zero cigarettes); (d) body mass index (kg/m^2^) following the technique reported in the WHO Cardiovascular Survey Methods (the WHO Manual) [10]; (e) subscapular skinfold thickness (in mm) following the technique reported in the WHO Manual [10]; (f) arm circumference (in mm) following the technique reported in the WHO Manual [10] with the crude measurement cleaned for the contribution of subcutaneous tissue using a formula that included the tricipital skinfold thickness [11]; (g) systolic blood pressure (mmHg) measured in supine position at the end of a physical examination, using a mercury sphygmomanometer, following the technique reported in the WHO Manual [10] having the average of two measurements taken one minute apart as the analytical variable; (h) heart rate (beats/min) derived from a resting ECG; (i) vital capacity (in L/m^2^) following the technique reported in the WHO Manual [10] having the best of two attempts used for analysis; (j) serum cholesterol (mg/dL) measured in casual blood samples following the technique by Anderson and Keys [12]; (k) urine glucose (yes = 1; no = 0) measured by urine dipsticks (uristix) in casual urine samples, definite presence used as a proxy of diabetes; (l) prevalence of major cardiovascular diseases at entry (0 = no; 1 = yes) as defined by the SCS criteria [13].

Baseline data were collected in 1960 before the era of the Helsinki Declaration and acceptance was implied with participation at entry examinations, while only later was oral informed consent obtained in view of collecting follow-up data.

### 2.4. Statistical Analysis

A descriptive statistic was computed for death rates of the CVD end-points during 61 years and Kaplan–Meier curves were produced for each CVD group.

A simplified description of mortality trends was created computing death rates for each CVD group in 3 time-blocks of about 20 years whose denominator was made by subjects alive at the beginning of each time-block. For each CVD group, age at death was computed.

Cox proportional hazards models were solved with 3 cardiovascular end-points and 12 risk factors as covariates. For each end-point, calibration was computed (proportion of cases in quintile classes of estimated risk).

To test the possible competing risks, the Fine–Gray procedure was used to challenge CHD mortality versus all other causes of death (that is, all causes other than CHD). Two models were solved, one with CHD as the primary event and the other causes as secondary event, another the other way round, both as a function of the risk factors previously used in Cox models.

Mean levels of baseline risk factors significantly predicting at least one CVD end-point were computed in men who died from any CVD in 3 subsequent time-periods of about 20 years and compared by ANOVA. Statistical significance was set at *p* value ≤ 0.05.

## 3. Results

In the 61-year follow-up, among the 1712 men examined at baseline, there were 1708 deaths (99.8%), while 3 men were still alive with an age range of 102 to 106 years and 1 man was lost to follow-up after 50 years when he was aged 91 years. All types of CVD deaths covered 46% of total mortality while those considered for this analysis were 93% of all CVD deaths (N = 727). Among them, 38.7% were diagnosed as CHD, 31.6% as stroke and 28.5% as HDUE (Table 1).

**Figure 1:**
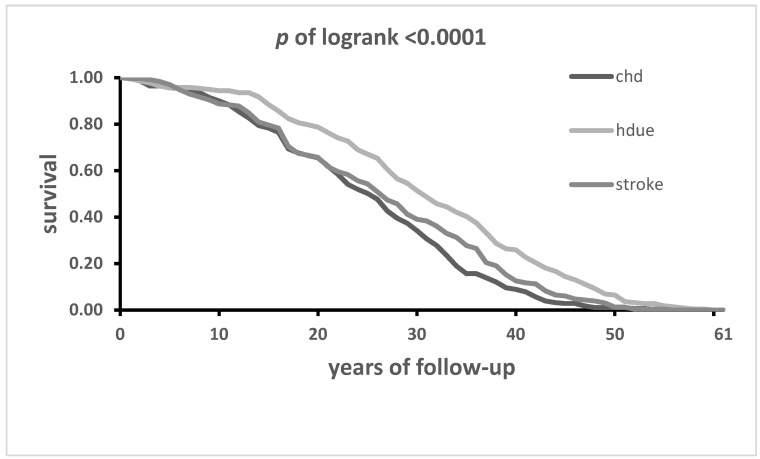
Lifelong Kaplan–Meier curves of CHD, stroke and HDUE until practical extinction of the 1712 men of the IRA cohort of whom 1708 were dead after 61 years.

Kaplan–Meier curves showed a highly statistically significant difference (*p* < 0.0001) among the three components of CVD mortality with a clear-cut and constant rightward displacement of the lifetime HDUE curve as compared to those of CHD and stroke (Figure 1).

**Figure 2:**
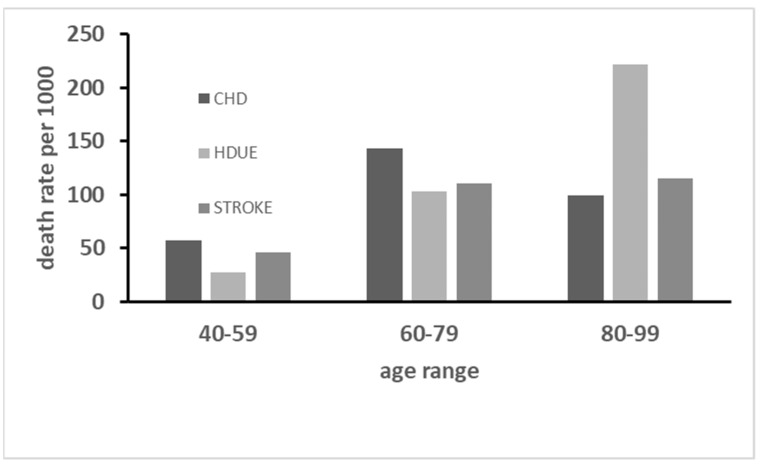
Death rates from CHD, HDUE and stroke in 3 time-periods (each of 20 years with age ranges at respective starting points) up to 61 years of follow-up. The *p* of chi^2^ across the three age ranges for each end-point is <0.0001.

On the other hand, the time-trends of the three major conditions showed large differences in the 20-year periods covering the whole follow-up. In fact, CHD death rate sharply increased between periods 1 and 2, but largely decreased in period 3 losing its first rank position; death rates for stroke increased from period 1 to period 3, maintaining its second rank position; while death rates for HDUE continuously increased becoming the first ranking in the third time period (Figure 2). All this suggested that HDUE, beyond other characteristics, had a different natural history compared with the other two major CVD categories. Moreover, age at death was largely different across the CVD groups, i.e., 73.2 (±19.4) years for CHD, 75.2 (±10.6) years for stroke and 79.7 (±10.8) years for HDUE (ANOVA *p* < 0.0001).

Mean levels of the selected risk factors at entry examination are reported in Table 2 where those related to the whole study population reflect the situation for men in Italian rural environments in the mid of last century. Among them, there were relatively high levels of blood pressure and cigarette consumption and relatively low levels of serum cholesterol. Prevalence of family heart attack was rather high but the vague term used in the questionnaire was preferred to a more technical term (like myocardial infarction) that could be difficult to understand those times.

Baseline mean levels of risk factors across CVD mortality groups did not show significant differences, except for heart rate (*p* = 0.0075) and serum cholesterol (*p* = 0.0066).

Cox models showed a variety of combinations of significant risk factors for CHD, stroke and HDUE as based on hazard ratios and their 95% confidence intervals (Table 3, Table 4 and Table 5). In particular, significant risk factors for CHD were age, cigarette smoking, systolic blood pressure, serum cholesterol and vital capacity; for stroke, age, systolic blood pressure, serum cholesterol, urine glucose and vital capacity; for HDUE, age, cigarette smoking, systolic blood pressure and heart rate. Significant risk factors common to the three CVD end-points were only age and systolic blood pressure. Among risk factors used in these models, we found a relatively high correlation and a collinearity problem between BMI and subscapular skinfold but, after removal of the latter, the new multivariable coefficients of risk factors were not significantly different from those of the original models.

The performance of the Cox models was estimated through the calibration procedure, reported in Table 6 for the three CVD end-points. Calibration was poor for all end-points since there were no clear trends in the proportion of cases distributed in quintile classes of estimated risk. We tried to find an explanation for the poor performance of the Cox models and Table 7 may contribute, at least partially. Using only risk factors with significant coefficients in at least one CVD end-point, mean levels were reported for those who died from any end-point in each time period of about 20 years. A definite trend occurred from time period 1 to time period 3, compatible with early deaths among those carrying unhealthy levels.

Table 8 reports on competing risks by Fine–Gray procedure whereby serum cholesterol was the critical risk factor in competition between CHD and other causes with a large positive and significant coefficient in the first model and a negative non-significant coefficient in the second model. On the other hand, age played a different role with positive higher level of coefficient in the second model.

## 4. Discussion

This study, based on a follow-up of 61 years in a cohort of middle-aged men, showed large differences across mortality from three major CVD conditions, i.e., CHD, HDUE (mostly composed of CHF) and stroke that related to several aspects of natural history and prediction; thus, suggesting that different diseases might be implicated and that etiology, mainly of HDUE, is still largely unknown but different from CHD. Although it was authoritatively suggested to base conclusions more on effect size, direction, plausibility, consistency, repeatability and clinical or practical utility rather than statistical significance [14], there are occasions, like the opposite sign (and thus direction) seen in this study for the predictive capacity of cholesterol versus CHD or other causes of death in the competing risk approach (Table 8), that deserve special attention as a statistical divergence, which is an important result presented. There are, moreover, several other characteristics that were different across the three CVD mortality end-points used in this analysis, that is, death rates, mortality time-trends, age at death, baseline levels of some risk factors and long-term risk factor predictive power. In particular, CHD was the most common condition. It declined in the last 20 years of follow-up, while during the same period there was a sharp increase in HDUE (including in large proportions CHF) mortality. Age at death was significantly lower for CHD, higher for HDUE and intermediate for stroke. The latter result in the IRA cohorts was recently confirmed in another 10 cohorts of the SCS [15].

Baseline mean levels of serum cholesterol were higher for those who died from CHD, lower for those who died from HDUE and intermediate for those who died from stroke, while it was significantly predictive (with a positive coefficient) of CHD events and stroke events and not predictive for HDUE events. In particular, a further novel finding in this analysis was the significant positive coefficient of serum cholesterol predicting stroke mortality since in previous analyses on shorter follow-up periods this was not found [16]. It is possible that in the late years, more thrombotic events added up but this conclusion remains uncertain since in the majority of cases we were unable to segregate thrombotic from hemorrhagic stroke.

All these important differences explicitly point to the fact that when comparing CHD with HDUE we are probably looking at different diseases, which might also be concluded if CHD is compared, using the Fine–Gray model that takes competition into account, to all other causes of death whereby a major risk factor such as cholesterol has a divergent role. As a consequence, it may not be appropriate to pool those three conditions into a single CVD end-point, simply because they relate to the same anatomical–physiological system although sharing the role of age and systolic blood pressure as significant risk factors, as is commonly but inappropriately performed in composite end-point analyses in the current literature. A Cox model including all CVD conditions (not reported in detail) may make sense in view of identifying risk factors that probably share at least some of the various end-points and for general preventive purposes. However, this does not help to improve the understanding of possible etiologies of the single conditions that, for certain aspects, remain different diseases.

The hypothesis was made of an important explanatory contribution of competing risks, a concept already documented on the same material using the 50-year follow-up data [17,18]. In other words, those with high cholesterol (and possibly other risk factors) are hit by a CHD event at a younger age while the others (relatively protected) become more exposed to other types of events that finally occur, but years later and at older ages. This likely explains part of the significant rightward displacement of the Kaplan–Meier curve for HDUE as compared to CHD and stroke (Figure 1) and the different age at death mainly comparing CHD with HDUE.

Some coefficients of major risk factors were smaller than in models run in the past for shorter follow-up period [7,16]. This can be explained by the fact that during the first periods subjects with higher levels were eliminated opening the chance to people with lower levels of risk factors to reach the disease status. In fact, the poor performance of the Cox models could partly be explained by the attrition phenomenon consisting in the early drop-out of subjects with worse risk factor levels. As a consequence, those dying later were usually carrying better levels of risk factors, contributing to dilute the magnitude of multivariable coefficients. This seems a universal phenomenon but probably enhanced by the lifelong follow-up that we have here.

Changes in risk factors level during the follow-up might also influence the multivariable coefficients. There has certainly been a sharp decline in cigarette smoking, a definite increase in blood pressure and a sizeable increase in serum cholesterol, but from previous analyses, we know that the baseline risk factor levels were predictive for at least 40 years when tested in partitioned blocks of follow-up time [19].

It was almost impossible to find studies in the literature that were really comparable to this one in terms of follow-up duration, practical extinction of the cohort and dealing with the same groups of CVD mortality with well-defined groups based on the international classification of diseases. The only “key word” providing somewhat similar analyses and references was “lifetime CVD risk”. Many studies claim to have estimated the lifetime risk of CHD but almost all have simply applied a lifetime Framingham risk score to their data [20]. Actually, they do not deserve a direct mention since they simply produced theoretical estimates applying an external score to their baseline data.

On the other hand, the Framingham score [20] was based on the use of total and HDL cholesterol, systolic and diastolic blood pressure, smoking habits and body mass index, all transformed into a variable number of discrete classes. The lifetime exposure was actually stopped at the age of 95 years and the overall death rate of the observed cohort was about 30%. Moreover, the assumption was made that those reaching the age of 75 years could be considered as “dead” although the majority of them died later. In fact, the concept of “lifetime” was interpreted in a substantially arbitrary way and the analytical approach had nothing to do with our approach where a cohort was followed-up until practical extinction (99.8%).

Other contributions based on the Framingham study dealing with the same general problem were mainly devoted to numerical estimates of the lifelong CVD incidence–mortality based on incomplete follow-up [21], including a specific analysis dedicated to CHF (in people without and with previous myocardial infarction) [22] and another to stroke [23]. The same authors made comparisons of 10 years versus lifelong events prediction [24,25], concluding that a new specific approach should be developed.

A huge analysis was conducted in the US pooling several large studies with data collected from 1964 through 2008 and exploiting the predictive role of blood pressure, smoking habits, diabetes and serum cholesterol and posing the age limit of 95 years. The outcome, defined by a pool of different major CVD, was predicted with largely different probabilities depending on the combination of high-low levels of the considered risk factors [26].

A Swedish study based on a risk factor population screening of 92,000 adults aged 40, 50 and 60 years, covered a maximum follow-up of 25 years [27]. Measured risk factors were serum cholesterol, smoking habits, blood pressure, body mass index, physical activity, self-reported health, education and marital status, all considered in discrete classes and combining them to determine three different risk profiles. The end-points were the occurrence of CHD, stroke, CHF and a pool of CVD. There was a coherence between the risk profiles and the so-called lifetime risk of CHD, but not for stroke and CHF, suggesting that this study had some similarity with our findings. However, the authors did not comment on this aspect and simply said that the distinction between specific CVD events was not a relevant problem since the real problem is the prevention through action on risk factors. This conclusion has a practical sense, but excludes any attention for possible differences across various CVD types and their possible etiology, similar to what we tried to achieve here.

A Chinese study published in 2015 involved almost 22,000 adults aged 35–84 where a few risk factors were measured and the follow-up was extended for 18 years [28], with pooled CVD events as the end-point. The estimate of lifetime CVD risk, with a limit posed at the age of 80 years, showed the ability of a few risk factors to segregate high from low risk for CVD events.

In three large meta-analyses run on the same population studies, but separately exploring different risk factors, it was impossible to identify a subgroup of common heart diseases, outside CHD (that is somewhat similar to those we call HDUE) since they were usually pooled with other vascular diseases preventing the possibility of carefully describing their characteristics [29,30,31].

It is clear that genomic analyses may improve long-term risk prediction and some contributions start to be of great interest [32], but this aspect could obviously not be considered in our data collected in the middle of the last century.

Finally, we have more recently investigated, in the context of 10 cohorts of 9063 middle-aged men of the Seven Countries Study followed for 60 years to extinction, whether competing risks of CHD mortality versus other causes of death, comparatively assessed by Fine–Gray versus Cox models, might help in explaining why regions with initially high serum cholesterol have higher mortality from CHD and lower from stroke and HDUE, while the reverse is found for those with initially lower serum cholesterol [33]. There were five important conclusions that lend support to the results presented here: (1) CHD mortality is bound to different risk factor relationships when put in competition with all other causes of death and with the sum of HDUE and stroke; (2) competing risk analyses (using Fine–Gray methods) are needed to assess risk factor capabilities in specific outcome types of CVD since they are different depending on outcome types considered (CHD versus HDUE plus stroke); (3) there are important differences among predictive covariates, in terms of the presence/absence and opposition of the coefficients’ algebraic sign when the inversion is performed of the primary versus the secondary outcome in the case of Fine–Gray, what is much less evident or absent in the Cox model which points to the need for a certain power, namely the number of enrolled individuals, in order to assess these aspects; (4) the changing algebraic sign of country coefficients, treated in the study as dummy variables, comparing direct versus inverse models might suggest a possible “ecologic“ interpretation of the Fine–Gray analysis; (5) it is clear that individual outcomes of CVD should not be summed-up or equated, which should be considered in all future investigations.

## 5. Limitations

A major strength of the present study is the practical extinction of the original cohort and the extremely long duration of follow-up that, with the detailed diagnosis of outcome results, has few if no comparative examples in the literature. However, these results cannot be extended to women who were not included since at the beginning of the Seven Countries Study it was estimated that the low incidence of heart disease would have required too large groups to be enrolled, unacceptable financial and organizational requirements in early sixties. Along these lines, the medical management and prognosis have changed significantly over this long follow-up period, limiting the relevance of the study results to modern practice. However, if one wishes to follow-up life-long middle-aged men, starting nowadays, the recruitment in order to have current medical management as the standard of potential treatment would certainly mean waiting another 65 years before presenting relevant results. Therefore, some degree of missed parallelism should necessarily be accepted between standard care and the need of following people for a life-long period, if one wishes today to have 60 or more years of follow-up. The same holds true for dipstick glycosuria as a marker for diabetes that is very crude and inaccurate. Nevertheless, the use of uristicks was decided because in the sixties another reliable, quick and standardized measurement of blood glucose to be made in field activities was not available. The performance of this procedure, at least from the time-trend of Table 7, is not so bad. Using the clinical diagnosis of diabetes based on history (instead of uristick) in multivariate models was not better (unpublished result).

We only took risk factors at baseline into consideration and evidently the risk factor profile is likely to have changed significantly during such a long follow-up period. We have previously addressed this issue in a recent paper [34]: it was concluded that “spontaneous long-term changes in major coronary risk factor levels were associated with changes in the same direction of coronary heart disease mortality risk modelled by the Weibull distribution, expressing a kind of natural experiment with an outcome that matches those of controlled preventive trials.”. On the other hand, what we call HDUE may be seen as a heterogenous group that might have silent ischemia as a pathophysiological thread ending up with CHF. However, real silent ischemia is an extremely rare condition [35], whereas we have fully documented that HDUE may represent a new definition of heart failure for epidemiological studies with important anatomical–pathological differences from real CHD cases as documented from the sixties [36].

## 6. Conclusions

It is extremely important to underline that composite end-point analyses of CVD mortality have the inherent risk of distorting the relations among factors and outcomes to the extent that, at least, CHD versus HDUE (or also possibly the sum of HDUE and stroke) and versus all other causes of death should be considered, not mixing them up for a composite end-point analysis of CVD, as is mostly performed in the literature. Moreover, there might be predictive differences in the analyzed covariates when a cohort presents around 39% of CHD deaths versus lower or higher proportions, which also depends, among other factors, on the respective proportions of stroke and HDUE. This fact has important consequences for epidemiology, public health and clinical practice since the potentially different etiology between CHD and HDUE (largely composed of CHF) points to the need of dissecting these causes in large ongoing and future investigations also separating stroke from the other components of CVD [36] and certainly not addressing composite end-points just to unduly increase the power of studies which is performed more than often [37], and may have unpredictable consequences depending on the analyzed cohorts.

The present study shows large differences in natural history and risk factors when comparing the three CVD conditions (CHD, HDUE and stroke), potentially indicating different etiologies and pointing to the need of not mixing them up in a grouped CVD category.

## Figures and Tables

**Table 1:** Death rates in 61 years from all-causes and selected CVD in 1712 men first examined in 1960.

Causes of Death	N	Rate per 1000	Proportion % within Selected Groups of CVD
All causes	1708	998	----
All cardiovascular diseases CVD	785	459	----
**Selected sub groups of CVD**			
Coronary heart disease	281	164	38.7
Stroke	230	134	31.6
Heart disease of uncertain etiology	216	126	29.7
Pool of selected groups of CVD	727	425	100.0

**Table 2:** Mean values at entry examination of selected risk factors distributed in the initial sample and in three classes of CVD mortality.

Risk Factor	Overall (N = 1712)	CHD	Stroke	HDUE	*p* of ANOVA
Age, years	49.1 (5.1)	49.0 (5.1)	49.7 (5.0)	49.5 (5.2)	0.4144
Family heart attack (0–1), % (*)	40.3 (1.8)	40.9 (2.9)	40.4 (3.2)	39.4 (3.3)	0.9380 (**)
Cigarettes smoked, N/day	8.7 (0.5)	8.9 (9.4)	7.4 (8.8)	8.0 (8.9)	0.1627
Body mass index, kg/m^2^	25.2 (3.7)	25.8 (3.7)	25.3 (3.7)	25.0 (3.7)	0.0579
Subscapular skinfold, mm	11.8 (5.8)	12.7 (6.2)	12.1 (6.3)	11.4 (5.7)	0.0664
Arm circumference, mm	268.6 (23.6)	270.0 (23.1)	269.6 (23.6)	268.7 (22.8)	0.8503
Systolic blood pressure, mmHg	143.6 (21.0)	146.4 (21.8)	145.0 (21.9)	142.7 (20.5)	0.1638
Heart rate, beats/min	71.3 (12.9)	71.7 (12.7)	71.6 (12.4)	68.4 (13.7)	**0.0075**
Vital capacity, L/m^2^	1.65 (0.24)	1.63 (0.25)	1.63 (0.23)	1.67 (0.24)	0.1736
Serum cholesterol, mg/dL	201.6 (40.8)	209.6 (42.2)	204.4 (38.8)	198.0 (39.3)	**0.0066**
Urine glucose (0–1), % (*)	5.1 (0.8)	4.6 (1.3)	6.1 (1.6)	4.6 (1.4)	0.7071 (**)
CVD prevalence (0–1), % (*)	4.7 (0.8)	4.6 (1.3)	4.8 (1.4)	4.6 (1.4)	0.9958 (**)

ANOVA across the three CVD mortality end-points; (*) proportions % and (standard error); (**) Chi-squared test; Significance in bold.

**Table 3:** Cox model for CHD deaths (N = 281) in 61 years as a function of 12 risk factors.

Variable	Delta	HR	95% CI	*p* of Coefficient
Age	5	1.41	1.23–1.61	**<0.0001**
Family heart attack	1	1.18	0.87–1.50	0.1814
Cigarettes smoked	9	1.18	1.06–1.32	**0.0034**
Body mass index	4	1.16	0.91–1.48	0.2232
Subscapular skinfold	6	0.95	0.78–1.15	0.6067
Arm circumference	24	0.87	0.74–1.03	0.1135
Systolic blood pressure	20	1.36	1.20–1.54	**<0.0001**
Heart rate	13	0.99	0.99–1.13	0.9396
Vital capacity	0.24	0.84	0.73–0.96	**0.0094**
Serum cholesterol	40	1.27	1.13–1.42	**<0.0001**
Urine glucose	1	1.06	0.60–1.90	0.8335
CVD prevalence	1	1.50	0.85–2.64	0.1619

Units of measurement as from Table 2. HR = hazard ratio; 95% CI = confidence intervals. Delta for computation of HR roughly corresponding to 1 standard deviation for continuous variables. Significance in bold.

**Table 4:** Cox model for stroke deaths (N = 230) in 61 years as a function of 12 risk factors.

Variable	Delta	HR	95% CI	*p* of Coefficient
Age	5	1.71	1.50–2.03	**<0.0001**
Family heart attack	1	1.14	0.87–1.49	0.3323
Cigarettes smoked	9	1.03	0.90–1.18	0.6414
Body mass index	4	0.95	0.72–1.26	0.7200
Subscapular skinfold	6	0.97	0.78–1.22	0.8092
Arm circumference	24	0.95	0.80–1.16	0.6730
Systolic blood pressure	20	1.28	1.10–1.48	**0.0009**
Heart rate	13	1.05	0.91–1.22	0.4740
Vital capacity	0.24	0.85	0.73–0.98	**0.0306**
Serum cholesterol	40	1.17	1.02–1.34	**0.0203**
Urine glucose	1	1.82	1.04–3.19	**0.0370**
CVD prevalence	1	1.51	0.81–2.81	0.1912

Units of measurement as from Table 2. HR = hazard ratio; 95% CI = confidence intervals. Delta for computation of HR roughly corresponding to 1 standard deviation for continuous variables. Significance in bold.

**Table 5:** Cox model for HDUE deaths (N = 216) in 61 years as a function of 12 risk factors.

Variable	Delta	HR	95% CI	*p* of Coefficient
Age	5	2.34	1.98–2.77	**<0.0001**
Family heart attack	1	1.17	0.89–1.54	0.2663
Cigarettes smoked	9	1.21	1.06–1.38	**0.0052**
Body mass index	4	0.95	0.70–1.30	0.7612
Subscapular skinfold	6	0.95	0.75–1.22	0.7014
Arm circumference	24	0.91	0.74–1.11	0.3426
Systolic blood pressure	20	1.36	1.16–1.58	**<0.0001**
Heart rate	13	0.81	0.69–0.96	**0.0145**
Vital capacity	0.24	0.92	0.78–1.09	0.3329
Serum cholesterol	40	1.07	0.93–1.23	0.3467
Urine glucose	1	1.72	0.89–3.32	0.1037
CVD prevalence	1	1.74	0.91–3.33	0.0950

Units of measurement as from Table 2. HR = hazard ratio; 95% CI = confidence intervals. Delta for computation of HR roughly corresponding to 1 standard deviation for continuous variables. Significance in bold.

**Table 6:** Calibration of Cox models with proportion of total cases distributed in quintile classes of estimated risk (Q1, Q2, Q3, Q4, Q5), for 3 CVD end-points.

End-Point	Q 1	Q2	Q3	Q4	Q5	Ratio Q5/Q1	*p* of Chi-Squared
Coronary heart disease	16.0	16.3	21.0	24.2	22.4	1.4	0.4298
Stroke	16.5	18.7	22.6	21.3	20.9	1.3	0.8498
Heart disease of uncertain etiology	19.0	17.1	18.0	21.8	24.1	1.3	0.8576

**Table 7:** Mean level of baseline risk factors significantly predicting at least one CVD end-point in men who died from any CVD in subsequent time periods.

Variable	Time Period 1 Year 0 to 20		Time Period 2 Year 21 to 40		Time Period 3 Year 41 to 61		*p* of ANOVA
	Mean	SD	Mean	SD	Mean	SD	
Age	51.6	4.9	49.3	4.7	44.6	3.2	**<0.0001**
Cigarettes smoked	9.2	9.5	8.8	9.3	4.9	7.4	**<0.0001**
Systolic blood pressure	155.9	25.6	141.8	17.4	134.1	15.5	**<0.0001**
Heart rate	74.3	14.8	69.7	12.0	67.7	10.7	**<0.0001**
Vital capacity	1.55	0.25	1.66	0.23	1.75	0.22	**<0.0001**
Serum cholesterol	210.6	40.6	205.6	40.3	190.7	38.2	**<0.0001**
Urine glucose *	7.3	2.1	4.5	1.2	1.7	1.2	**0.0626** **

Unit of measurement as from Table 2; * proportion (%) and standard error; ** *p* of chi squared.

**Table 8:** Fine–Gray models for the evaluation of competing risks of CHD versus other causes (all other than CHD) mortality as a function of 12 risk factors.

	CHD (as Primary) vs. OTHER CAUSES					OTHER CAUSES (as Primary) vs. CHD					
Risk Factor	Coefficient	*p* Value	HR	95% CI		Coefficient	*p* Value	HR	95% CI		*t*-Test
Age	−0.0150	0.2390	0.93	0.82	1.05	0.0461	<0.0001	1.26	1.19	1.34	−4.31 **
Family heart attack	0.1244	0.3102	1.13	0.89	1.44	−0.0069	0.9044	0.99	0.89	1.11	0.97
Cigarettes smoked	0.0035	0.5592	1.03	0.93	1.15	0.0066	0.0258	1.06	1.01	1.12	−0.46
BMI ^$^	0.0210	0.5170	1.09	0.84	1.40	0.0052	0.7414	1.02	0.90	1.16	0.44
Subscapular skinfold	0.0053	0.7582	1.03	0.84	1.26	−0.0138	0.1012	0.92	0.83	1.02	1.00
Arm circumference	−0.0011	0.7486	0.97	0.83	1.15	−0.0024	0.1488	0.94	0.87	1.02	0.34
Systolic blood pressure	0.0063	0.0490	1.13	1.00	1.29	0.0014	0.4296	1.03	0.96	1.11	1.32
Heart rate	−0.0038	0.4354	0.95	0.84	1.08	0.0037	0.1444	1.05	0.98	1.12	−137
Vital capacity	−0.2883	0.2984	0.93	0.82	1.06	−0.1117	0.4124	0.97	0.91	1.02	-0.57
Serum cholesterol	0.0042	0.0042	1.18	1.05	1.33	−0.0009	0.1972	0.96	0.91	1.02	3.13 *
Urine glucose	−0.0736	0.8104	0.93	0.51	1.70	0.1772	0.2342	1.19	0.89	1.60	−0.74
CVD prevalence	0.0464	0.8776	1.05	0.58	1.89	−0.0040	0.9840	1.00	0.70	1.41	0.15

Units of measurement as from Table 2. HR: hazard ratios (with delta as used in Table 3, Table 4 and Table 5); *t*-test = test T between coefficients of the two models; ^$^ = corresponding coefficients in absence of subscapular skinfold and arm circumference, separately considered (10 degrees of freedom instead of 12) due to collinearities with BMI, were, respectively: 0.02264 (*p* = 0.160, HR 1.09, 95% CI 0.96–1.24) and −0.01991 (*p* = 0.03, HR 0.92, 95% CI 0.86–0.99), whereas the results for the remaining covariates were globally unchanged. ** *p* < 0.0001; * *p* = 0.0018.

## Data Availability

The original data are not publicly available. However, research projects are evaluated centrally by an ad hoc committee.
